# Valvular Heart Disease in Non-Valvular Heart Failure Continuum: The Role of Cardiopulmonary Exercise Testing

**DOI:** 10.3390/biomedicines13102415

**Published:** 2025-10-02

**Authors:** Kiriaki Mavromoustakou, Michail Botis, Panagiotis Iliakis, Ioannis Leontsinis, Panagiotis Xydis, Kyriakos Dimitriadis, Christina Chrysohoou, Konstantinos Tsioufis

**Affiliations:** First Department of Cardiology, School of Medicine, National and Kapodistrian University of Athens, Hippokration General Hospital, 115 27 Athens, Greece; mavromoustakoukiriaki@yahoo.gr (K.M.); mgmpotis94@gmail.com (M.B.); giannisleontsinis@gmail.com (I.L.); panosxydis@yahoo.gr (P.X.); dimitriadiskyr@yahoo.gr (K.D.); chrysohoou@usa.net (C.C.); ktsioufis@gmail.com (K.T.)

**Keywords:** heart failure, cardiopulmonary exercise testing, valvular heart disease, valvular regurgitation

## Abstract

**Background/Objectives**: Patients with non-valvular heart failure frequently develop valvular disease. However, the prevalence of valvular disease across patients with different heart failure etiologies remains underexplored. This study aimed to investigate the burden of VHD among patients with non-valvular heart failure, and secondly evaluate its association with cardiopulmonary test. **Methods**: We analyzed data from patients with non-valvular heart failure (HF) who were evaluated as outpatients at the HF clinic between February 2020 and November 2024. Patients were categorized into three groups: coronary artery disease-related HF (CAD-HF), dilated cardiomyopathy (DCM), and other causes (e.g., hypertension, diabetes, and various cardiomyopathies). Demographic and clinical characteristics, as well as echocardiographic and cardiopulmonary exercise testing (CPET) results, were evaluated. **Results**: Among all groups mild mitral regurgitation (MR) was the most common valvular disease, followed by mild tricuspid regurgitation (TR). Patients with CAD-HF frequently had mild aortic regurgitation (AR) compared to DCM (23.6% vs. 14.9%, *p* = 0.05). In the CPET subgroup, which included 41 patients who consented to participate, in patients with moderate-to-severe VHD had significantly lower VO_2_/HR (oxygen pulse), VO_2_max, and OUES, indicating worsened functional capacity despite similar left ventricular ejection fraction. Hypertension and atrial fibrillation were independently associated with greater valvular disease severity on multivariable analysis. **Conclusions**: No significant differences in valvular disease between patients with DCM and CAD-HF were documented, apart from a higher prevalence of mild AR in the CAD-HF group. Patients with moderate-to-severe valvular regurgitation demonstrated worse cardiopulmonary performance, regardless of ejection fraction, highlighting the important role of CPET in evaluating the functional impact of valvular heart disease in this population.

## 1. Introduction

Heart failure and valvular heart disease are pathophysiologically interrelated, exhibiting a bidirectional and dynamic interaction [1]. Although valvular heart disease is a well- established cause of heart failure, it is also frequently observed among patients with other heart failure etiologies, contributing to increased symptom burden and adverse prognosis [2,3]. The management of valvular heart disease in this context, particularly mitral and tricuspid regurgitation, has been rigorously investigated [4,5,6,7]. However, data that comprehensively document the prevalence, characteristics, and clinical impact of valvular comorbidities in heart failure of non-valvular origin remain scarce, representing an important gap in the current evidence.

Utilizing data from a contemporary cohort at a tertiary care hospital, we aimed to systematically delineate the burden of valvular heart disease among patients with non-valvular heart failure. We subsequently stratified our findings according to heart failure etiology, distinguishing between ischemic, dilated, and other causes, including hypertension and various cardiomyopathies. Within the subgroup of patients with coronary artery disease, we further investigated the severity of valvular heart disease across the spectrum of left ventricular ejection fraction. Additionally, we explored the association between valvular disease severity and powerful cardiovascular disease predictors, including atrial fibrillation, diabetes mellitus, and hypertension.

Consensus statements and prospective clinical trials have underscored the role of cardiopulmonary exercise testing (CPET) in the evaluation of patients with heart failure, as it contributes prognostic insights across the entire spectrum of the disease and aids therapeutic decisions, particularly in the context of advanced heart failure, where valvular heart disease tends to be more frequent [8,9,10,11]. However, the classification of CPET response according to valvular disease severity in this population is yet to be established. Hence, we analyzed key CPET-derived parameters, including oxygen pulse (VO_2_/HR), maximal oxygen uptake (VO_2_max), oxygen uptake efficiency slope (OUES), metabolic equivalents (METs), and end-tidal carbon dioxide pressure (PETCO_2_), comparing patients with moderate-to-severe valvular impairment to those with only mild valvular involvement. This approach aimed to quantify the functional impact of valvular heart disease within the spectrum of non-valvular heart failure.

## 2. Materials and Methods

### 2.1. Setting

Consecutive patients were evaluated at the Outpatient Heart Failure Clinic of Hippokration General Hospital in Athens, Greece, between February 2020 and November 2024. All research was conducted according to a prespecified protocol, adhering to the principles of the Declaration of Helsinki. All participants voluntarily provided informed consent before enrollment.

### 2.2. Sample

This is a retrospective cohort study from the medical records of the Outpatient clinic of our university hospital, where all consecutive patients who had been evaluated between February 2020 and November 2024, were enrolled. All research was conducted according to a prespecified protocol, adhering to the principles of the Declaration of Helsinki. All participants voluntarily had provided informed consent for anonymous use of their clinical data for scientific purposes. The sample consisted of 361 patients with chronic heart failure. Patients with heart failure secondary to valvular disease or without an echocardiographic assessment in their records or unable to provide a comprehensive medical history were excluded from this study.

### 2.3. Data Collection

Data collected for all patients included epidemiological factors (sex and age), comorbidities, medications, and family medical history (Table 1). Additionally, vital signs, including blood pressure, heart rate, oxygen saturation levels, and temperature, were recorded. Electrocardiogram, echocardiography, and cardiopulmonary exercise testing were also performed. The severity of valvular disease was standardized according to the EACVI (European Association of Cardiovascular Imaging) guidelines. The patients were categorized into three groups. Group 1 included patients with dilated cardiomyopathy (DCM), Group 2 consisted of patients with heart failure due to coronary artery disease, and Group 3 comprised patients with heart failure due to other causes, such as hypertension, diabetes mellitus, hypertrophic cardiomyopathy, and arrhythmogenic right ventricular (RV) cardiomyopathy.

### 2.4. Statistical Analysis

Normality of data distribution was assessed using the Kolmogorov–Smirnov test. Variables with a normal distribution were reported as mean ± standard deviation (SD), while non-normally distributed variables were expressed as median with interquartile range (IQR). Group comparisons for normally distributed data were performed using Student‘s *t*-test or one-way ANOVA, as appropriate. For skewed data, the Mann–Whitney U test or Kruskal–Walli’s test was applied. Categorical variables were presented as frequencies and percentages, and differences between groups were assessed using the Chi-square test. A two-tailed *p*-value of <0.05 was considered statistically significant. Multivariate logistic regression analysis and adjusted odds ratios were used to correlate the severity of valvular abnormalities and the presence of cardiovascular risk factors. The variables assessed significantly in univariate analysis were entered as independent variables in multivariate logistic regression analysis. All statistical analyses were performed using IBM SPSS Statistics, version 27 (IBM Corp., Armonk, NY, USA).

## 3. Results

A total of 361 patients were included in the analysis, with a mean age of 70.43 ± 11.63 years, consisting of 273 males and 88 females. Of these, 101 patients (28%) were categorized into Group 1; 68 (67.3%) had HFrEF, 9 (8.9%) had HFmrEF, and 24 (23.8%) had HFpEF (heart failure with recovered ejection fraction). Group 2 included 195 (54%), of whom 140 (71.8%) had HFrEF (LVEF ≤ 40%), 15 (7.7%) had HFmrEF (LVEF 41–49%), and 40 (20.5%) had HFpEF (LVEF ≥ 50%). Lastly, Group 3 consisted of 65 patients (18%) (50 due to Hypertension, 4 due to DM, 10 due to HCM and 1 due to ARVC). In this group, 43 (66.2%) had HFrEF, 6 (9.2%) had HFmrEF, and 16 (24.6%) had HFpEF (Table 1). A family history of DCM was present in 2 patients (2%) in Group 1 and a family history of CAD was present in 59 patients (30%) in Group 2.

The most common valvular diseases across all groups were mild and moderate mitral regurgitation (MR), mild aortic regurgitation (AR), and mild tricuspid regurgitation (TR). Specifically, 57 (56.4%) in Group 1, 115 (59%) patients in Group 2, and 38 (58.5%) in Group 3 had mild MR; 32 (32%) in Group 1, 79 (40.5%) in Group 2, and 27 (41.5%) in Group 3 had mild TR; 15 (14.9%) in Group 1, 46 (23.6%) in Group 2, and 14 (21.5%) in Group 3 had mild AR; 18 (17.8%) in Group 1, 40 (20.5%) in Group 2, and 6 (9.2%) in Group 3 had moderate MR (Table 2).

### 3.1. Most Common Valvular Diseases in Patients with DCM vs. Patients with CAD-HF

**Figure 1:**
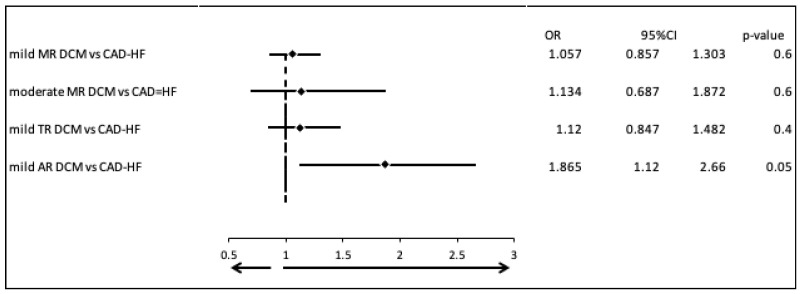
Most common valvular diseases in patients with DCM vs. patients with CAD-HF.

We examined the most common valvular diseases in patients with dilated cardiomyopathy (DCM; Group 1) compared to those with heart failure due to coronary artery disease (CAD-HF; Group 2). There were no clinically and statistically significant differences in the prevalence of mild mitral regurgitation (MR) (OR = 1.057, 95% CI: 0.857–1.303, *p* = 0.6), moderate MR (OR = 1.134, 95% CI: 0.687–1.872, *p* = 0.6), or mild tricuspid regurgitation (TR) (OR = 1.120, 95% CI: 0.847–1.482, *p* = 0.4). However, mild aortic regurgitation (AR) was found to be significantly more prevalent in the CAD-HF group (OR = 1.865, 95% CI: 1.120–2.660, *p* = 0.05) (Figure 1).

### 3.2. Comparison of Valvular Disease Patterns in DCM Patients with HFpEF/HFmrEF Versus Those with HFrEF

**Figure 2:**
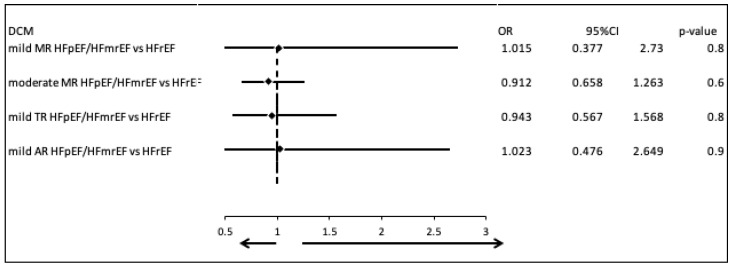
Comparison of valvular disease patterns in DCM patients with HFpEF/HFmrEF versus those with HFrEF.

We compared the most common valvular diseases between patients with DCM and HFpEF or HFmrEF, and those with DCM and HFrEF. There were no clinically and statistically significant differences in the prevalence of mild mitral regurgitation (MR) (OR = 1.015, 95% CI: 0.377–2.730, *p* = 0.8), moderate MR (OR = 0.912, 95% CI: 0.658–1.263, *p* = 0.6), mild tricuspid regurgitation (TR) (OR = 0.943, 95% CI: 0.567–1.568, *p* = 0.8), and mild aortic regurgitation (AR) (OR = 1.023, 95% CI: 0.476–2.649, *p* = 0.9) (Figure 2).

### 3.3. Comparison of Valvular Disease Patterns in CAD-HF Patients with HFpEF/HFmrEF Versus Those with HFrEF

**Figure 3:**
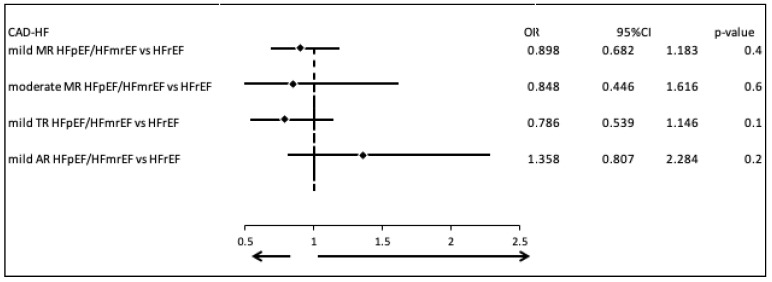
Comparison of valvular disease patterns in CAD-HF patients with HFpEF/HFmrEF versus those with HFrEF.

We evaluated the prevalence of the most common valvular diseases in patients with CAD-HF, comparing those with HFpEF or HFmrEF to those with HFrEF. There were no statistically or clinically significant differences in the occurrence of mild mitral regurgitation (MR) (OR = 0.898, 95% CI: 0.682–1.183, *p* = 0.4), moderate MR (OR = 0.848, 95% CI: 0.446–1.616, *p* = 0.6), mild tricuspid regurgitation (TR) (OR = 0.786, 95% CI: 0.539–1.146, *p* = 0.1), or mild aortic regurgitation (AR) (OR = 1.358, 95% CI: 0.807–2.284, *p* = 0.2) (Figure 3).

### 3.4. Correlation of CPET with Most Valvular Diseases in Patients with DCM and CAD-HF

Cardiopulmonary exercise testing (CPET) was performed in 41 patients with DCM and CAD-HF (Table 3). We compared VO_2_/HR (oxygen pulse), VO_2_max (maximal oxygen uptake), OUES (oxygen uptake efficiency slope), METs (metabolic equivalents) and PETCO_2_ (end tidal partial pressure of CO_2_) between patients with moderate-to-severe valvular regurgitation and those with only mild valvular involvement. The mean ejection fraction in patients with mild valvular disease and those with moderate to severe valvular disease was 36% ± 5% and 33% ± 4%, respectively. The mean difference in PETCO_2_ between the two groups was 3.033 (mean of moderate/severe VD 41.34 mmHg, mean of mild VD 38.33 mmHg, 95% CI: −2.876 to 9.895, *p* = 0.6) and METs was 1.34 (mean of moderate/severe 4.55 METs, mean of mild VD 5.90 METs, 95% CI: −0.067 to 2.760, *p* = 0.06) indicating no statistically and clinically difference, while the mean difference in OUES was 756.3 (mean of moderate-severe and mild valvular regurgitation was 1376.5 and 2132.8, respectively, 95% CI: 411.6 to 1100.9, *p* = 0.001), VO_2_/HR was 3.577 (mean of moderate/severe VD 13.26 mL O_2_/beat, mean of mild VD 16.83 mL O_2_/beat, 95% CI: 1.073 to 7.054, *p* = 0.05) and VO_2_max was 4.32 (mean of moderate/severe VD 15.86 mL/kg/min, mean of mild VD 20.18 mL/kg/min, 95% CI: 0.23 to 8.41, *p* = 0.03) reflecting both statistical and clinical difference.

### 3.5. Univariate and Multivariable Logistic Regression Analysis

**Figure 4:**
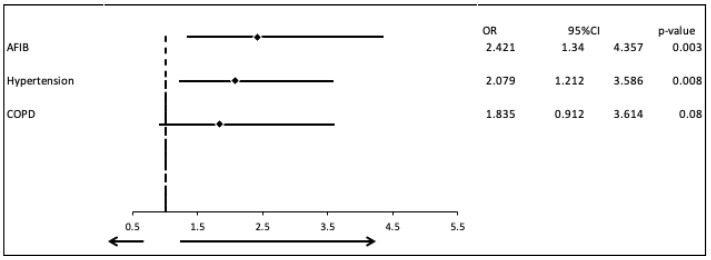
Multivariate logistic regression analysis including the covariates Atrial fibrillation Hypertension and COPD.

In the univariate logistic analysis, the covariates of hypertension, and atrial fibrillation were correlated significantly with the severity of the valvular diseases in patients with CAD-HF and DCM (OR = 2.118, 95% CI: 1.249–3.609, *p* = 0.005 and OR = 2.458, 95% CI: 1.378–4.364, *p* = 0.002, respectively). Therefore, a multivariable logistic analysis was performed, which confirmed these associations, with OR =2.079 for hypertension (95% CI: 1.212–3.586, *p* = 0.008) and OR =2.421 for atrial fibrillation (95% CI: 1.340–4.357, *p* = 0.003) (Table 4, Figure 4).

## 4. Discussion

This study demonstrates that the severity of valvular heart disease, in the context of non-valvular heart failure, does not significantly differ across distinct heart failure etiologies. However, increased valvular disease severity is associated with significantly impaired cardiopulmonary performance, as evidenced by lower OUES, reduced VO_2_/HR and diminished VO_2_max. Traditional cardiovascular disease risk factors, including diabetes mellitus, hypertension and dyslipidemia, are independently associated with increased burden of valvular heart disease, in this population.

Previous research has shown that mitral regurgitation frequently coexists with heart failure [12,13]. The valvular disease patterns observed in our study have shown that mild MR was the most common valvular abnormality, occurring in 59% of patients with CAD-HF and 56.4% of patients with DCM. There were no significant differences in the prevalence of MR between patients with DCM and CAD-HF, suggesting that they may share similar valvular pathology, probably regarding mitral valve insufficiency. However, aortic regurgitation (AR) prevalence was significantly higher in the CAD-HF group (23.6%) compared to the DCM group (14.9%). There are no previous studies to support our findings regarding AR and the small size of the subgroup should be taken into consideration; therefore, further investigation and research with a larger sample are needed. TR was the second most common valvular disease in both groups, with no significant difference. Interestingly, aortic stenosis, which is the most common valvular heart disease in the general population in developed countries, is not that common in our sample [14]. One explanation for this finding is that we included patients with heart failure not primarily caused by valvular disease. Although aortic stenosis and CAD-HF or DCM can coexist, our results suggest that when present, aortic stenosis likely predates and leads to heart failure, rather being a secondary finding, therefore meeting our exclusion criteria.

In patients with DCM, progressive dilation of the ventricles can lead to functional tricuspid and mitral valve regurgitation, which further reduces the ejection fraction. Additionally, in patients with CAD-HF, left ventricular remodeling and papillary muscle dysfunction can cause MR and a reduction in EF, while right ventricular ischemia and RV dilation due to overload can lead to TR [15,16]. The correlation of EF and prevalence of valvular diseases was investigated in all groups with no significant differences. These findings do not align with existing knowledge, so larger studies are needed for additional research into this correlation. It is crucial to highlight that in patients with DCM, 23.8% of patients had EF within normal range, which most likely reflects recovered EF, due to medication or other interventions.

In recent years, cardiopulmonary exercise testing (CPET) has been a method for evaluating patients with heart failure, providing information about prognosis and assessing the progress of a patient [17,18]. CPET can identify asymptomatic patients with exertion limitations and also patients with impaired exercise capacity who may benefit from earlier valve repair or replacement [19]. In this study, we investigate the correlation of end-tidal carbon dioxide pressure (PETCO_2_), VO_2_/HR (oxygen pulse), VO_2_max (maximal oxygen uptake), OUES (oxygen uptake efficiency slope) and METs (metabolic equivalents) which represent functional capacity, ventilatory efficiency, and cardiopulmonary function, with the severity of valvular disease in patients with CAD-HF and DCM. Our results suggest that there is a correlation between valvular severity and worsened functional capacity, but not with ventilatory inefficiency. It is crucial to underline that both groups had approximately equal ejection fractions; hence, CPET is superior for evaluating these patients. These findings highlight the need for further investigation and support the usefulness of CPET for the evaluation of patients after transcatheter valve procedures [9].

In the univariate analysis, hypertension and atrial fibrillation were significantly associated with increased valvular heart disease burden and in the multivariate model, hypertension and atrial fibrillation were identified as independent predictors of greater valvular disease severity [20]. Studies have shown that hypertension and atrial fibrillation are not only common risk factors in heart disease, but also independent predictors of valvular dysfunction. Additionally, patients with valvular heart disease and atrial fibrillation have an increased risk of adverse outcomes in mitral and tricuspid valve pathology [21,22].

### Strengths and Limitations of the Study

There are several limitations of this study. This study is a single-center study with an observational design, therefore lacks randomization and limit generalizability. Moreover, the sample size of the CPET group and patients with other causes of HF was relatively small, therefore type II statistical error cannot be excluded. A larger sample size would strengthen the generalizability of our findings and allow more robust stratification by HF phenotype (HFrEF, HFmrEF, HFpEF). Furthermore, adjunctive parameters including ventricular dimensions and global longitudinal strain, which may provide insight into ongoing ventricular remodeling and serve as predictors of valvular involvement, were not systematically evaluated. Apart from the lack of global longitudinal strains measurements, there were no measurements derived from cardiac magnetic resonance in our study that could aid us in quantifying valvular disease burden, which is an area of high interest in future trials. Finally, valvular disease severity was assessed only by transthoracic echocardiography and not confirmed by other methods such transesophageal echocardiography.

## 5. Conclusions

This study shows that the severity of valvular heart disease is similar in patients with dilated cardiomyopathy (DCM) and those with heart failure due to coronary artery disease (CAD-HF), with the exception of mild aortic regurgitation, which was more common in the CAD-HF group. Moreover, greater valvular regurgitation severity was significantly correlated with impaired cardiopulmonary performance as assessed by CPET parameters. These findings suggest that CPET could be a valuable tool for assessing functional limitations in this patient group. However, larger studies are needed to confirm these results.

## Figures and Tables

**Table 1:** Baseline clinical characteristics.

	DCM (*n* = 101)	CAD-HF (*n* = 195)	Other (*n* = 65)	*p*-Value
HFrEF, *n* (%)	68 (67.3%)	140 (71.8%)	43 (66.2%)	0.6
HFmrEF, *n* (%)	9 (8.9%)	15 (7.7%)	6 (9.2%)	0.9
HFpEF, *n* (%)	24 (23.8%)	40 (20.5%)	16 (24.6%)	0.6
Age (mean ± SD)	72 ± 4	73 ± 2	69 ± 5	0.04
Female, *n* (%)	28 (27.7%)	43 (22.1%)	16 (24.6%)	0.5
Male, *n* (%)	73 (72.3%)	152 (77.9%)	49 (75.4%)	0.5
DM, *n* (%)	28 (27.7%)	79 (40.5%)	13 (20%)	0.03
Hypertension, *n* (%)	30 (29.7%)	146 (74.9%)	50 (76.9%)	0.001
Dyslipidemia, *n* (%)	20 (19.8%)	140 (71.8%)	40 (61.5%)	0.001
AF, *n* (%)	27 (26.7%)	43 (22.1%)	19 (29.2%)	0.3
COPD, *n* (%)	13 (12.9%)	35 (17.9%)	8 (12.3%)	0.4

AF: atrial fibrillation, CAD-HF: coronary artery disease heart failure, COPD: chronic obstructive pulmonary disease, DCM: dilated cardiomyopathy, DM: diabetes mellitus, HFmrEF: heart failure with mildly reduced ejection fraction, HFpEF: heart failure with preserved ejection fraction, HFrEF: heart failure with reduced ejection fraction.

**Table 2:** Valvular disease among study population.

	DCM (*n* = 101)	CAD-HF (*n* = 195)	OTHER (*n* = 65)	*p*-Value
Mild AR, *n* (%)	15 (14.9%)	46 (23.6%)	14 (21.5%)	0.2
Moderate AR, *n* (%)	5 (5%)	11 (5.6%)	1 (1.5%)	0.3
Severe AR, *n* (%)	0	0	0	
Mild MR, *n* (%)	57 (56.4%)	115 (59%)	38 (58.5%)	0.8
Moderate MR, *n* (%)	18 (17.8%)	40 (20.5%)	6 (9.2%)	0.1
Severe MR, *n* (%)	1 (1%)	1 (0.5%)	0	0.6
Mild TR, *n* (%)	32 (32%)	79 (40.5%)	27 (41.5%)	0.6
Moderate TR, *n* (%)	8 (8%)	17 (8.7%)	4 (6.2%)	0.4
Severe TR, *n* (%)	5 (5%)	9 (4.7%)	1 (1.5%)	0.5
Mild PR, *n* (%)	0	2 (1%)	0	0.2
Moderate PR, *n* (%)	0	0	0	
Severe PR, *n* (%)	0	0	0	
Mild AS, *n* (%)	0	4 (2.1%)	2 (3.1%)	0.06
Moderate AS, *n* (%)	0	5 (2.6%)	4 (6.2%)	0.05
Severe AS, *n* (%)	0	0	0	
Mild MS, *n* (%)	0	2 (1%)	1 (1.5%)	0.56
Moderate MS, *n* (%)	0	0	0	
Severe MS, *n* (%)	0	0	0	
Mild TS, *n* (%)	0	0	0	
Moderate TS, *n* (%)	0	0	0	
Severe TS, *n* (%)	0	0	0	
Mild PS, *n* (%)	0	0	0	
Moderate PS, *n* (%)	0	0	0	
Severe PS, *n* (%)	0	0	0	

AR: aortic regurgitation, AS; aortic stenosis, CAD-HF: coronary artery disease heart failure, DCM: dilated cardiomyopathy MR: mitral regurgitation MS: mitral stenosis, PS: pulmonary stenosis, PR: pulmonary regurgitation, TR: tricuspid regurgitation, TR: tricuspid stenosis.

**Table 3:** CPET group valvular diseases.

	DCM (*n* = 19)	CAD-HF (*n* = 22)	(*n* = 41)
Mild AR, *n* (%)	5 (26.3%)	3 (13.6%)	8
Moderate AR, *n* (%)	1 (5.3%)	0	1
Severe AR, *n* (%)	0	0	0
Mild MR, *n* (%)	9 (47.4%)	16 (72.7%)	25
Moderate MR, *n* (%)	3 (15.8%)	1 (4.5%)	4
Severe MR, *n* (%)	1 (5.3%)	0	1
Mild TR, *n* (%)	6 (31.6%)	9 (40.9%)	15

AR: aortic regurgitation, CAD-HF: coronary artery disease-related heart failure, DCM: dilated cardiomyopathy, MR: mitral regurgitation, TR: tricuspid regurgitation.

**Table 4:** Multivariable logistic analysis, including the covariates gender, age, T2 diabetes mellitus, hypertension, dyslipidemia, COPD and atrial fibrillation.

	Univariate Analysis				Multivariate Analysis			
	OR	95% C.I.			OR	95% C.I.		
		Lower	Upper	*p*-Value		Lower	Upper	*p*-Value
Gender	1.00	0.96	1.023	0.826				
Age	1.00	0.993	1.009	0.8				
Diabetes Mellitus	1.322	0.770	2.255	0.306				
Hypertension	2.118	1.249	3.609	0.005	2.079	1.212	3.586	0.00801
Dyslipidemia	0.809	0.480	1.367	0.429				
COPD	1.747	0.888	3.350	0.097	1.835	0.912	3.614	0.08229
Atrial Fibrillation	2.458	1.378	4.364	0.002	2.421	1.340	4.357	0.00318

C.I: confidence interval, COPD: chronic obstructive pulmonary disease, OR: odds ratio.

## Data Availability

The data presented in this study are available on request from the corresponding author.

## Abbreviations

AFIB	Atrial fibrillation
AR	Aortic regurgitation
AS	Aortic stenosis
CAD-HF	Coronary artery disease-related HF
CI	Confidence interval
COPD	Chronic obstructive pulmonary disease
CPET	Cardiopulmonary exercise testing
DCM	Dilated cardiomyopathy
HF	Heart failure
HFmrEF	Heart failure mildly reduced ejection fraction
HFpEF	Heart failure preserved ejection fraction
HFrEF	Heart failure reduced ejection fraction
HR	Heart rate
METs	Metabolic equivalents
MR	Mitral regurgitation
MS	Mitral stenosis
OR	Odds ratio
OUES	Oxygen uptake efficiency slope
PETCO2	End-tidal carbon dioxide pressure
PR	Pulmonary regurgitation
PS	Pulmonary stenosis
TR	Tricuspid regurgitation
TS	Tricuspid stenosis
VHD	Valvular heart disease
VO_2_max	Maximal oxygen uptake
